# A symbiotic aphid selfishly manipulates attending ants via dopamine in honeydew

**DOI:** 10.1038/s41598-021-97666-w

**Published:** 2021-09-17

**Authors:** Tatsumi Kudo, Hitoshi Aonuma, Eisuke Hasegawa

**Affiliations:** 1 Laboratory of Animal Ecology, Faculty of Agriculture, Hokkaido University, Sapporo, 060-8589 Japan; 2 Research Institute for Electronic Science, Hokkaido University, Sapporo, 060-0812 Japan; 3 Laboratory of Animal Ecology, Graduate School of Agriculture, Hokkaido University, Kita 9, Nishi 9, Sapporo, 060-8589 Japan; 4 Present Address: Department of Biology, Graduate School of Science, Kobe University, 1-1 Rokkodai-cho, Nada-ku, Kobe, 657-8501 Japan

**Keywords:** Evolution, Evolutionary ecology, Behavioural ecology

## Abstract

Symbiotic relationships are widespread in nature, but the mechanisms maintaining these relationships remain to be elucidated because symbiosis incurs a maintenance cost to each participant, which lowers its reproductive rate. In host-parasite relationships, parasites are known to manipulate the host's behavior selfishly, and there is an arms race between them. Selfish manipulations also occur in symbiosis, but the effects of selfish manipulations on symbiosis are not fully understood. Here, we show that an ant-associated aphid manipulates attending ants to receive stronger protection. Aphid honeydew regurgitated by ants contains dopamine (DA). The ants showed low aggressiveness before contact with the aphids, but it rose after contact. Administration of DA to the ants increased ant aggressiveness as the concentration increased, while an antagonist of DA inhibited this effect. The other 3 amines showed no effect on aggressiveness. A previous study showed that attending ants selfishly manipulate aphids by increasing the reproductive rate of green morph to obtain high-quality honeydew. These results suggest that mutual selfish manipulation benefits both participants and is likely to strengthen symbiosis. The selfishness of each participant may contribute to sustaining this symbiosis because their selfishness increases their long-term fitness.

## Introduction

Amazing biodiversity is found in nature^1^, and how and why it is maintained is one of the most important issues in ecology and evolutionary biology^2^. However, under natural selection, symbiotic relationships are predicted to decay because the cost necessary to maintain the relationship lowers the instantaneous reproductive rate (= arithmetic mean fitness)^3^. This cost should invite cheaters that do not pay the cost but exploit benefits from the symbiotic system. Despite the inevitable invasion by cheaters, why are many symbiotic relationships maintained?

The exclusion of cheaters from cooperative systems, including symbiosis, has been explained by the following two mechanisms: (1) partner choice^4^ (do not choose cheaters as partners) and (2) sanctions^5^ (penalizing cheaters). However, there is a third possibility that symbiotic relationships can be maintained by mutual selfish manipulations, i.e., selfish manipulations of each participant to the other may reinforce the symbiotic relationships^6^.

Selfishbehavioralmanipulation in biological relationships between different species is widespread from parasitism to mutualism^7–11^. Host-parasite relationships are thought to lead arms races that increase the costs incurred by the hosts^12^. While in symbiosis, mutual selfish manipulations can increase mutual dependency because such manipulations increase benefits of participants from the symbiotic system. In such case, selfish manipulations may be accepted easily by a partner in symbiosis^6,13^.

One example of this prediction is ant-acacia symbiosis^6^. Extrafloral nectar secreted by acacia contains a chemical that inhibits the enzyme activity necessary for sucrose digestion in ants. The acacia simultaneously secretes a sugar that can be digested by attending ants that had lost the ability of sucrose digestion^6^. As the manipulated ants cannot live without this sugar from the acacia, the ants must strongly defend the acacia^6^. In this system, selfish manipulation by the host is beneficial to both participants, and thus this manipulation is acceptable to the ants^6^.

The color polymorphic aphid *Macrosiphoniella yomogicola* has green and red morphs in Hokkaido^14^. The main attending ant, *Lasius japonicus*, also undergoes a symbiosis in which the ants protect the aphids from predators and the aphids provide honeydew to the ants. In this symbiosis, *L. japonicus* prefers the green morph, which has a lower reproductive rate (likely due to its high nutritional investments to honeydew) than the red morph^15^and increases the reproductive rate of the green morph to an equal level to that of the red morph^15^. The ants do not predate the nutritionally inferior red morph^14^, unlike in another ant-aphid symbiosis in which *L. japonicus* workers feed on aphid individuals that secrete less honeydew than other individuals^16^. Thus, in the symbiosis focused in this study, the ants intentionally maintain the coexistence (another manipulation) of both morphs^14,15^. Although the reason for this second manipulation is not known, it is apparent that ants selfishly manipulate the green morph to obtain more resources.

In another case, secretions from a lycaenid caterpillar change amine levels in the attending ants’ brains to increase the aggressiveness of the ants^11^. Several previous studies have shown that any of 4 amines (dopamine: DA, serotonin: 5HT, tyramine: TA, and octopamine: OA) in the ants’ brains can mediate the behavior of the ant workers^17–21^. *M. yomogicola* may selfishly manipulate attending ants to receive stronger protection by secreting honeydew to control these amines in the brain, like lycaenid caterpillars, although aphids were previously thought to be a kind of domestic animals for attending ants^22^.

The purpose of this study is to examine whether *M. yomogicola* selfishly manipulates attending-ants' aggressiveness to receive stronger protection. For this purpose, the amines in ant-collected and directly collected honeydew of *M. yomogicola* are measured, and the effect of each amine on the aggressiveness of the ants are tested. Based on the results, we will discuss the role of mutual manipulation in the evolution of stronger mutual dependency in this symbiosis.

## Results

**Figure 1:**
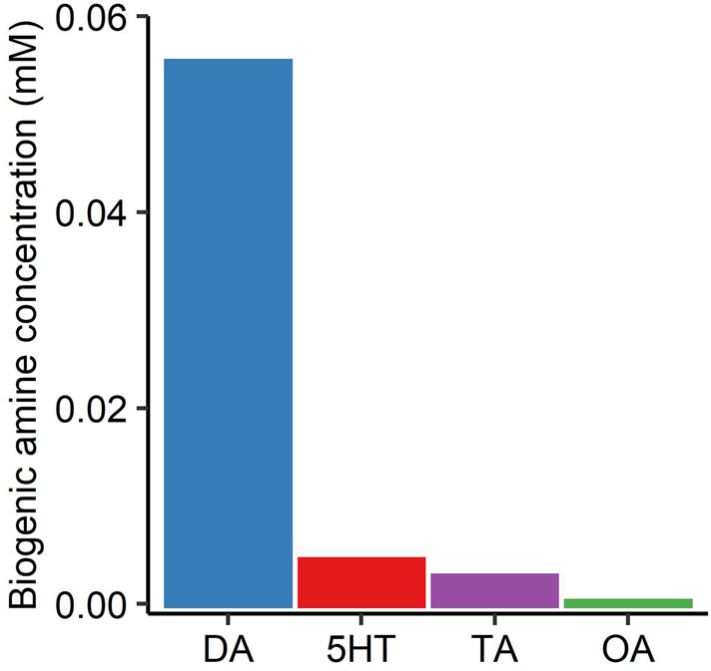
Concentrations of 4 biogenic amines in the ant-collected honeydew of *M. yomogicola* (total volume of honeydew = 6.56 µl, 0.111 ± 0.0118 µl (mean ± S.E.) per ant, n = 59). The concentrations of dopamine (DA), serotonin (5HT), tyramine (TA), and octopamine (OA) were 5.52 × 10^−2^, 4.36 × 10^−3^, 2.70 × 10^−3^, and 1.09 × 10^−4^ mM, respectively.

We collected a total of 6.56 µl (0.111 ± 0.0118 µl (mean ± S.E.) per ant, n = 59; 1.09 ± 0.231 µl per colony, n = 6) of ant-collected honeydew from the crops of 59 ants attending six colonies of *M. yomogicola* (occurring on shoots of mugwort). All samples have been summed and measured to obtain a robust result without measuring errors from the small sample amounts. Four biogenic amines and 11 related chemicals (precursors or metabolites) were detected in the total ant-collected honeydew (for details, see Supplementary Table S1 online). Figure 1 shows the concentrations of the 4 biogenic amines in the total ant-collected honeydew. The concentration of DA, 5HT, TA, and OA of the ant-collected honeydew was 5.52 × 10^–2^, 4.36 × 10^–3^, 2.70 × 10^–3^, and 1.09 × 10^–4^ mM, respectively.

In addition, we directly collected the honeydew from the aphids by two methods (see Methods section). DA was detected in 1 out of the 6 samples (35.6 µM) using method 1, and 7 out of 20 samples (2.39 × 10^2^, 88.1, 31.2, 29.2, 30.9, 28.5, 28.8 nM of honeydew dissolved in 10 µl of distilled water; see Methods) in the method 2. As the detection limit of the LC/MS is ca. 4.5 pg/µl (= 29.4 nM), "non-detected" does not mean absence of DA. Thus, we tested the mean concentration of the above detected samples and it is significantly different from zero (Wilcoxon singed-rank test: V = 28, n = 7, *p* = 0.016). It is concluded that at least the aphids on the above shoots secreted DA in their honeydew.

**Figure 2:**
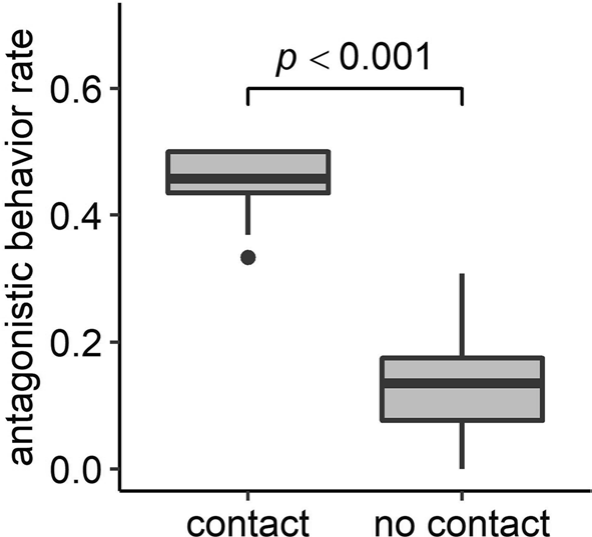
Rates of antagonistic behaviors of “contact” ants (ants attending aphids) and “no-contact” ants (ants climbing the stem from the ground to an aphid colony). The rates were significantly different between the contact group and the no-contact group (GLMM: estimate =  − 1.608, n = 80, z =  − 18.165, *p* < 0.001). The boxplots show the median, upper and lower quartiles, minimum and maximum, and any outliers.

Figure 2 shows that the frequency of antagonistic behavior of the ants that have been on aphid colonies (contact) was significantly higher than that of the ants collected at the base of colonized shoots while climbing up to the aphid colonies (no contact) (a generalized linear mixed model (GLMM) setting ant colony as a random effect: estimate = − 1.608, n = 80, z = − 18.165, *p* < 0.001). The result indicated that the attending ants become aggressive after contact with the aphids.

**Figure 3:**
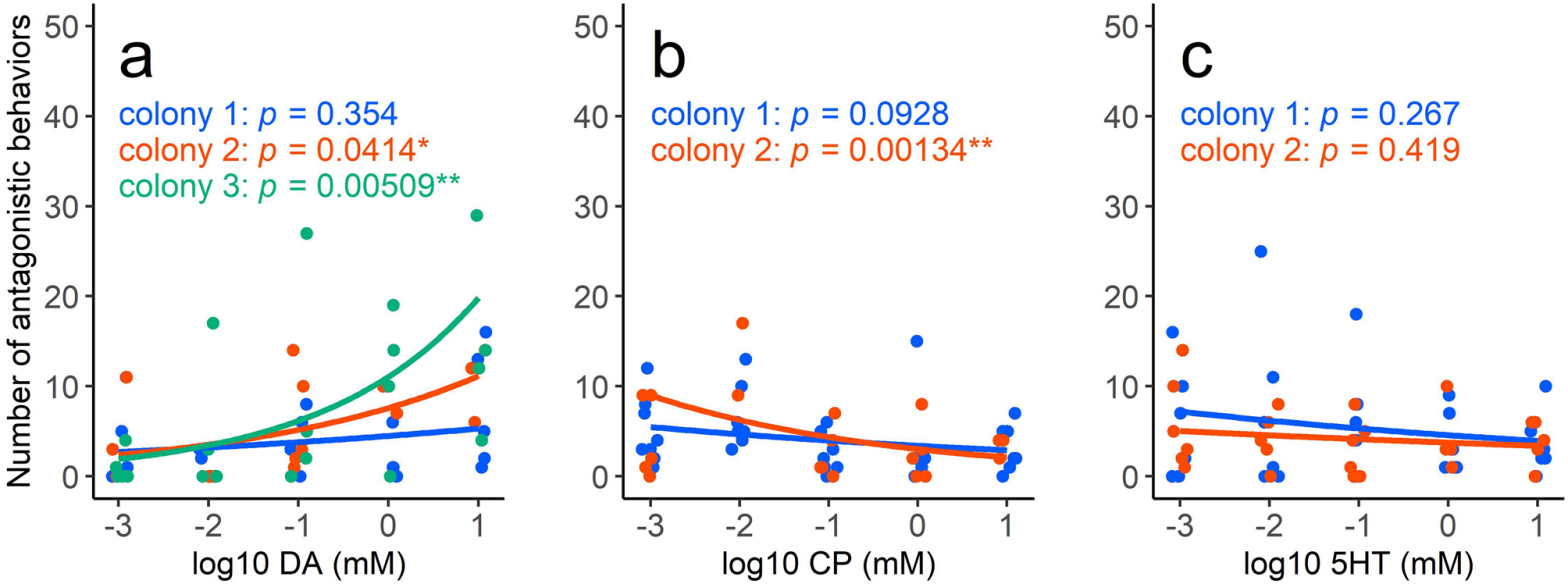
Relationship between the total number of antagonistic behaviors and the concentration of (**a**) DA (colony 1: y = exp (1.503 + 0.1680x), colony 2: y = exp (2.028 + 0.3843x), colony 3: y = exp (2.403 + 0.5839x) ), (**b**) CP (colony 1: y = exp (1.224 − 0.1594x), colony 2: y = exp (1.110 − 0.3617x)), and (**c**) 5HT (colony 1: y = exp (1.522 − 0.1509x), colony 2: y = exp (1.323 − 0.0979x)). The regression lines (blue curves) estimated by a generalized linear model (GLM) with negative binomial distribution for each ant colony. Colony variation was explained by a generalized linear mixed model (GLMM) setting ant colony as a random effect (see Table 1). *P*-values for the slopes were shown and asterisks indicated statistically significant differences from zero (likelihood-ratio test; **p < 0.01, *p < 0.05). The plots were slightly horizontally shifted for easier viewing.

Table 1 and Fig. 3a–c show the effect of DA, an antagonist of DA, Chlorpromazine (CP), and 5HT on the aggressiveness of *L. japonicus* workers. Aggressiveness toward a predator of the aphids (a ladybug, *Harmonia axyridis*, from the same host plant) increased with the concentration of administered DA (Table 1 upper: GLMM setting ant colony as a random effect; estimate = 0.370, n = 60, z = 3.194, *p* = 0.0014). Chlorpromazine significantly inhibited the effect of 10 mM DA (Table 1 middle: GLMM; estimate = − 0.176, n = 59, z = − 2.120, *p* = 0.034). Serotonin (5HT) showed no significant effect (Table 1 lower: GLMM; estimate = − 0.121, n = 58, z = − 1.288, *p* = 0.198). TA and OA resulted in no increase in the aggressiveness of the ants when compared with the effect of 10% sucrose solution (Wilcoxon rank sum test: TA vs 10% sucrose; W = 55.5, n = 26, *p* = 0.147: OA vs 10% sucrose; W = 97, n = 28, *p* = 0.973). Thus, it is concluded that DA mediates the observed increase in the aggressiveness of *L. japonicus* workers.

**Table 1:** The results of generalized linear mixed model analysis by setting aphid colony as a random effect for 3 experimental chemicals.

Administrated chemical	Effect		Estimate	S.E	z	*p*
DA (n = 60)	Fixed	Intercept	2.0041	0.1972	10.161	< 0.001***
		log10 (concentration)	0.3701	0.1159	3.194	0.0014**
	Random	Ant colony	S.D. = 4.673 × 10^−6^			
CP (n = 59)	Fixed	Intercept	1.20729	0.14901	8.102	< 0.001***
		log10 (concentration)	− 0.17560	0.08282	− 2.120	0.034*
	Random	Ant colony	S.D. = 2.355 × 10^−7^			
5HT (n = 58)	Fixed	Intercept	1.44401	0.16621	8.688	< 0.001***
		log10 (concentration)	− 0.12089	0.09388	− 1.288	0.198
	Random	Ant colony	S.D. = 2.286 × 10^−6^			

We consider that the significance of the intercept is not as important as the slope, so only significant *p*-value (*p* < 0.05) of the slope was bolded.

Dopamine (DA) showed a significant positive effect (estimate = 0.370, n = 60, z = 3.194, *p* = 0.0014) on the aggressiveness of *L. japonicus* workers as with its concentrations. An antagonist of DA (chlorpromazine: CP) significantly suppressed the effect of an enough DA concentration (10 mM) (estimate =  − 0.176, n = 59, z =  − 2.120, *p* = 0.034). Serotonin (5HT: the second-most concentrated amine in the honeydew in attending ant crops) had no effect (estimate =  − 0.121, n = 58, z =  − 1.288, *p* = 0.198). Both TA and OA had no significant effect on the aggressiveness of ants (the results for TA and OA are shown in the text).

***p < 0.001, **p < 0.01, *p < 0.05.

The mean number of antagonistic behaviors was compared before and after intake of 10% sucrose or 10% sucrose with NR. Both showed no effect on the antagonistic behaviors (Wilcoxon signed-rank test, 10% sucrose: V = 12, n = 12, *p* = 0.227; 10% sucrose with NR: V = 41.5, n = 15, *p* = 0.516). The results showed that NR and sucrose were not the cause of increased aggressiveness.

One may doubt DA in the ants' crops has been synthesized by the ants themselves. However, administration of the artificial honeydew significantly lowered all the aggression indices (vigilant behavior, aggressive behavior, and total (antagonistic behavior); for the definitions, see Methods; Wilcoxon signed-rank test for vigilant behavior: V = 772, n = 24, *p* = 0.0033; for aggressive behavior: V = 80, n = 24, *p* = 0.014; for antagonistic behavior: V = 782.5, n = 24, *p* = 0.002). Thus, DA synthesis by the ants themselves after taking honeydew is unlikely.

## Discussion

The ant-collected honeydew of *M. yomogicola* contained DA (5.52 × 10^–2^ mM: see Fig. 1 and Results section), and ants that came into contact with aphids showed significantly higher aggressiveness than no-contact ants from the same host plant (Fig. 2). A part of directly collected honeydew also contained DA (see Results). These observations could be explained by three possible hypotheses: (1) DA in the aphids’ honeydew increases ant aggressiveness, (2) satiety increases ant aggressiveness, and (3) ant aggressiveness increases during foraging independently of honeydew.

Hypotheses 2 and 3 can be rejected because aggressiveness did not significantly increase after intake of 10% sucrose solution or the artificial honeydew. The intake of the artificial honeydew instead lowered the aggressiveness of the ants. Thus, taking food without DA rather lowers the aggressiveness of the ant, and this is adaptive because waste of ants with resources in their crops by battles would lead a loss of foraging efficiency of their colony.

In addition, our results showed that (1) the increased aggressiveness of the ants was mediated by DA, (2) a DA antagonist (CP) suppressed the effect of DA (Fig. 3b and Table 1), and (3) 5HT, TA, and OA did not affect ant aggressiveness. Because the DA concentration in the ant-collected honeydew was 5.52 × 10^–2^ mM (Fig. 1), which was within the range tested in experiment 3 (10–0.001 mM), the concentration (or amount) of DA in the ant-collected honeydew would be sufficient to increase the ants’ aggressiveness. The concentrations of 5HT, TA and OA detected in the ant collected honeydew were lower than the tested ones, and thus we concluded that 5HT, TA, and OA in the ant-collected honeydew did not affect aggressiveness. Although the ant-collected honeydew was collected from the ants’ crops, the possibility of contamination was carefully excluded (see Methods) and DA was detected from the honeydew that was directly collected. The artificial honeydew experiment showed that aggressiveness did not increase when the ants were fed the artificial honeydew without DA. These results support the hypothesis that DA in honeydew increased the aggressiveness of the attended ants. Even if other chemicals affect ants’ aggressiveness, the hypothesis that DA in the aphid honeydew increases the ants’ aggressiveness cannot be rejected by our results. In conclusion, the possibility that aphids manipulate the behavior of ants through DA to receive stronger protection is supported by our results.

The above results suggest that the protection of aphids by ants is controlled by the aphids themselves. This conclusion contradicts the previous view that "ant-associated aphids are domestic animals of the attending ants"^22^. A previous study shows that *L. japonicus* increases the reproductive rate of the green morph of *M. yomogicola* (the more attractive morph for the attending ants), which has a lower reproductive rate than that of the red morph under no ant attendance^14^. This fact suggests that *L. japonicus* also selfishly manipulates *M. yomogicola* to obtain more nutrients. As a result, both species mutually conduct selfish manipulation to their partner in this symbiosis. However, these selfish manipulations are mutually beneficial, and if these manipulations disappear, the sustainability of the symbiosis would decrease.

In host-parasite relationships, hosts evolve to resist manipulation by parasites^23^. In such one-sided relationships, the host is likely to bear more costs than the parasites^24^, and this asymmetry could drive the evolution of counterstrategies in the host. This counteradaptation, in turn, leads to further adaptations in the parasites and antagonistic coevolution between them, i.e., an arms race^25^ or chase-away^26^. This is because the opponent’s manipulation results in a loss of fitness of the manipulated participant (= cost). However, in cases of symbiosis, selfish manipulations can lead to mutual benefits as shown in this study (Figs. 1, 2, and 3 and Table 1). In other words, mutual manipulation leads to a win–win relationship between the two participants with stronger mutual dependency.

The dependencies of *L. japonicus* and *M. yomogicola* are asymmetric because the aphids absolutely depend on attendance for survival (when the ants are removed, the aphids’ colony soon becomes extinct^14^), but the ants can depend on other resources^22^. Such an asymmetry in dependency would lead to strong attractiveness to the partner in the high-dependence participant. For example, an acacia plant detains attending ants by secreting extrafloral nectar that contains chemicals to enforce the ants to digest only the sugars in its own nectar^6^. This manipulation prevents the acacia from the loss of attendance. Similarly, *M. yomogicola* individuals that are at a weak position in this symbiosis may secrete the preferred nutrients for *L. japonicus* in their honeydew. Further studies will elucidate how aphids realize the required high dependency of ants to own by controlling ant behavior.

This study seems to be an example in which mutual selfish manipulations strengthen the maintenance of symbiosis, i.e., each dependency on the other is strengthened by the other’s selfish manipulation. Biological relationships can result in a win–win relationship because of the selfish evolution of each party, as suggested by this study. All organisms live in a community, and there are huge biological relationships. A win–win biological relationship should be sustainable for a long time, as both parties obtain fitness benefits from the relationship. However, each party should pay some costs to maintain the relationship (e.g., the investment in honeydew by ant-associated aphids or scale insects^27^ and ant cost for caring them^28^). Such “cooperative” relationships can be threatened by the invasions of cheaters, such as social parasites observed in social insects^29^. Invasions by cheaters are inevitable, as a free rider can exploit the costs paid by honest participants. Therefore, the next target is to elucidate how a cooperative system can remove invaded cheaters. The wonderful biodiversity in nature may be a key to answering this fascinating question. Unfortunately, community biology currently analyzes the dynamics of a community on a species basis^30^. However, adaptive evolution occurs on an individual (or allele) basis. Therefore, individual (or allele)-based analyses of the dynamics of communities would be required to understand the evolution and maintenance of biodiversity in nature.

## Materials and methods

### Materials

*Macrosiphoniella yomogicola* is a color-dimorphic (red or green) aphid that accompanies the mugwort *Artemisia montana* in Hokkaido. This mugwort forms a genet composed of many clonal shoots connecting to each other by roots. *M. yomogicola* colonies are always attended by several ant species^31^. We used aphid colonies attended by *Lasius japonicus* only, as this is the most frequent and specialized to care for this aphid^32^. The ladybug *Harmonia axyridis* is a major predator of this aphid. *L. japonicus* and *H. axyridis* used in the following experiments were collected in September–October 2019 and 2020 at the Sapporo campus of Hokkaido University and Makomanai Park in Sapporo, Hokkaido, Japan. They were kept in plastic cases at 25 °C. The ants were fed 10% honey until three days before the experiment, and the ladybug was fed turtle food, ReptoMin (Tetra, Melle, Germany). All experiments were performed in accordance with relevant guidelines and regulations.

### Experiment 1: Measurement of biogenic amines in ant-collected honeydew

We measured biogenic amines in honeydew of aphids using several ways. I) Stored honeydew in ant' crops were collected. In July 2019, we collected approximately 10 *L. japonicus* workers moving downward on a shoot from the aphid-colony. Honeydew was sampled from six aphid colonies on different shoots. Each ant's abdomen was pushed down slowly with the thumb on a slideglass. We collected regurgitated honeydew (ant-collected honeydew) by using a glass capillary tube (Microcaps 0.25 µl, Drummond Scientific Company, Pennsylvania, USA), and the ant-collected honeydew (total volume: 6.56 µl) was used in a measurement. To prevent contamination from ant tissues, nontransparent regurgitated honeydew was excluded. This method removed the variance among shoots, and thus statistical tests for differences in the concentrations of each amine was impossible. However, this method gave a more robust result for the 4 amine-concentrations because measurement error from small volumes of samples can be removed. In this study, confirmation of presence of amines is needed. A frozen sample was dissolved in a buffer (140 mmol l^–1^ NaCl, 10 mmol l^–1^ KCl, 6 mmol l^–1^ CaCl_2_, 2 mmol l^–1^ MgCl_2_, 44 mmol l^–1^ glucose, 2 mmol l^–1^ TES, pH 7.2). The dissolved honeydew was added to 50 µl of ice-cold 0.1 mol l^–1^ perchloric acid containing 5 ng of 3,4-dihydroxybenzylamine (DHBA; Sigma, St Louis, MO, USA) as an internal standard. After centrifugation (4 °C, 15,000 g, 30 min), 35 µl of supernatant was collected. Biogenic amines in the sample were measured using high-performance liquid chromatography (HPLC) with electrochemical detection (ECD). Details regarding the HPLC-ECD system are provided in previous studies^33–35^, but the detector potential was changed to 880 mV versus an Ag/AgCl reference electrode. II) To remove the suspicion that detected amines were synthesized by ants, we measured concentration of dopamine (DA; as this is the focal amine (see Results section)) in directly collected honeydew from the aphids. We measured honeydew collected two different ways, 1) in July 2021, we brought 20 shoots with the aphids to the laboratory. Ants were removed, and each shoot was put into a 50 ml centrifuge tube filled by water. Each shoot was checked by 15 min for 3 h, and when an aphid secreted honeydew at the tip of abdomen, we collected it by the capillary. We could collect 6 samples by this method, but the amounts of samples were very small (0.135 ± 0.252 µl (mean ± S.D.)). Thus, we collected honeydew by the 2nd method. When the shoots were overnighted honeydew dropped on the surface of the shoots, and we collected the dropped honeydew by wiping it by the capillary for each shoot. Honeydew was condensed due to evaporation and thus we could not absorb honeydew in the capillary. For the 20 shoots, we dissolved the collected honeydew into 10 μl of distilled water. All the samples were kept at − 80 ℃ until measurement. This additional measurement was conducted by a LC/MS (the detection limit is ca. 4.5 pg/µl) in the Global Facility Center of Hokkaido University.

### Experiment 2: Differences in ant aggressiveness before and after contact with aphids

We examined whether ants that had contact with the aphids increased in aggressiveness. In July 2019, we collected 2–7 ants attending an aphid colony (considered “contacted”) and those climbing the stem from the ground (considered “no-contact”). Each group from a shoot (the same ant colony) was placed in a petri dish (diameter: 57 mm, height: 16 mm) and left for one minute. After introducing a ladybug, the ants’ behaviors were recorded for 5 min using a digital video camera (HC-V720M, Panasonic, Japan). After the experiment, the petri dishes were wiped with 75% ethanol to prevent pheromonal effects. The ladybug used was changed in each experiment. We considered a contact to have occurred between an ant and the ladybug when the ant’s antennae touched the ladybug. We classified contact into three types: (1) aggressive behavior: spraying formic acid or biting; (2) vigilant behavior: the ant tapped the ladybug more than 4 times with its antennae; and (3) no reaction, leaving without any interaction after a touching. Aggressive and vigilant behaviors were summed as antagonistic behaviors because there were many ants that did not show aggressive behaviors. The experiment was repeated as for the numbers of ants for each shoot, and a total of 20 shoots were tested. No. of ants used is shown in each statistical result.

### Experiment 3: The effects of 4 amines and a DA antagonist

We examined the effects of the 4 amines and a DA antagonist on the aggressiveness of *L. japonicus* workers. For DA (dopamine hydrochloride, NACALAI TESQUE, INC., Kyoto, Japan), 5HT (serotonin–creatinine sulfate monohydrate, Wako, Osaka, Japan), and the DA antagonist (CP; chlorpromazine hydrochloride, Wako, Osaka, Japan), we prepared a dilution series (10, 1, 0.1, 0.01, 0.001 mM) of each chemical in 10% sucrose solution with a few amounts of neutral red (NR; Wako, Osaka, Japan), and the CP solution was adjusted to include 10 mM DA. The effects of 10 mM TA (tyramine hydrochloride, Wako, Osaka, Japan) and OA (octopamine hydrochloride, TOKYO CHEMICAL INDUSTRY, Tokyo, Japan) were also examined. Aggressiveness was measured for each ant, and it introduced into a 0.6 ml tube with 20 μl of each solution absorbed in a cotton ball (approximately 5 mm in diameter) placed in the inside of the cap. The tubes were left 24 h at 25 °C. The aggressiveness of the ant was recorded using the same method above. After recording, each ant was crushed on filter paper, and the intake of the chemical was confirmed by the red color. When intake was not confirmed, we removed such data from the analyses. To confirm the effects of 10% sucrose or NR, the same experiment was conducted using both solutions. In each experiment, 15 ants without contact with the aphids from 2 ~ 3 shoots (= ant colonies) were used.

### Experiment 4: Effects of sugars in aphid honeydew

To remove the possibility of DA synthesis by ants, we conducted another experiment in which the aggressiveness of the ants was compared before and after the intake of artificial honeydew. Our preliminary analysis showed that only sucrose and melezitose were detected in honeydew, and the latter is 2.3-fold higher in concentration (EH unpublished data). We made artificial honeydew (AH) with this sugar ratio by dissolving the two sugars in distilled water with a little NR. The aggressiveness of starved ants from two colonies (n = 24 for each) toward a ladybug was measured before the intake of the AH. Each ant was reared with 10 μl of AH for 30 min in a 0.6 ml tube. Then, we re-measured the aggressiveness of each ant toward the same ladybug. After the experiment, each ant was crushed on filter paper to confirm the intake of AH.

## Statistical analyses

For Experiment 2, we compared the rate of antagonistic behavior between the two treatments using a generalized linear mixed model (GLMM) with a binomial distribution and a logit link function. Dummy variables (no contact: 0, contact: 1) were set as a fixed effect, and the ant colony (ants collected from the same shoot) was set as a random effect. For Experiment 3, the regression of the antagonistic behaviors against the chemical dilution series was examined with a GLMM with a negative binomial distribution and log link function. The common logarithm of the concentration of the chemicals was set as a fixed effect, and ant colony was set as a random effect. The difference in the number of antagonistic behaviors before and after feeding on 10% sucrose was compared with Wilcoxon signed-rank test after normality checks with the Shapiro–Wilk test, and differences in variances were tested with F tests. The differences between ants fed 10 mM TA and those fed OA in 10% sucrose and between ants fed 10% sucrose solution with NR and without NR were compared with a Wilcoxon rank-sum test. For Experiment 4, differences in the antagonistic behaviors before and after taking AH were compared with Wilcoxon signed-rank test. All analyses were conducted by using R (ver. 4.0.0)^36^. We used the packages lme4^37^, MASS^38^ and exactRankTests^39^.

## Supplementary Information

Supplementary Information.

## Data Availability

The datasets used in the current study will be deposited on figshare.
