# Supplementary material for: A symbiotic aphid selfishly manipulates attending ants via dopamine in honeydew
Source: Sci Rep. 2021 Sep 17;11:18569. doi: 10.1038/s41598-021-97666-w (PMC8448758; doi:10.1038/s41598-021-97666-w)
Supplement: Supplementary file 1 — Supplementary Information. [file 41598_2021_97666_MOESM1_ESM.pdf]

Supplementary Information

A symbiotic aphid selfishly manipulates attending ants via dopamine in honeydew

Tatsumi Kudo[1†]*, Hitoshi Aonuma[2] and Eisuke Hasegawa[3]

1: Laboratory of Animal Ecology, Faculty of Agriculture, Hokkaido University, Sapporo 060-8589, Japan.

2: Research Institute for Electronic Science, Hokkaido University, Sapporo 060-0812, Japan

3: Laboratory of Animal Ecology, Graduate School of Agriculture, Hokkaido University, Sapporo 060-8589, Japan.

†: Present Address: Laboratory of Animal Ecology, Graduate School of Agriculture, Hokkaido University, Sapporo 060-8589, Japan.

*Corresponding Author: tatsu_cu@eis.hokudai.ac.jp

Supplementary Table S1. The biogenic amines, their precursors and metabolites in the ant-collected honeydew of *Macrosiphoniella yomogicola*. Total volume of the honeydew was 6.56 μl as the sum of 59 ants from 6 independent host shoots.

Name of amine, precursor or metabolite	Concentration in the ant-collected honeydew (mM)
3-methoxytyramine	1.30×10^{-2}
5-hydroxyindole acetic acid	4.09×10^{-4}
serotonin	4.36×10^{-3}
5-hydroxytryptophan	1.30×10^{-4}
dopamine	5.52×10^{-2}
3,4-dihydroxymandelate	2.66×10^{-2}
3,4-dihydroxyphenylacetic acid	3.64×10^{-3}
epinephrine	1.15×10^{-2}
N-acetylserotonin	1.36×10^{-2}
N-acetyldopamine	6.67×10^{-3}
N-acetyltyramine	9.29×10^{-5}
octopamine	1.09×10^{-4}
tyramine	2.70×10^{-3}
tryptophan	0.147
tyrosine	8.84×10^{-3}
